# Assessment of Novel Inhaler Technique Reminder Labels in Image Format on the Correct Demonstration of Inhaler Technique Skills in Asthma: A Single-Blinded Randomized Controlled Trial

**DOI:** 10.3390/ph14020150

**Published:** 2021-02-12

**Authors:** Iman Basheti, Bassam Mahboub, Laila Salameh, Mena Al-Ani, Ammar Abdulrahman Jairoun, Basema Saddik, Eman Abu-Gharbieh

**Affiliations:** 1 Department of Clinical Pharmacy and Therapeutics, Faculty of Pharmacy, Applied Science Private University, P.O. Box 166, Amman 11931, Jordan; dr_iman@asu.edu.jo; 2 Rashid Hospital, Dubai Health Authority, Dubai 4545, United Arab Emirates; BHMahboub@dha.gov.ae (B.M.); lisalameh@dha.gov.ae (L.S.); 3 Sharjah Institute for Medical Research, University of Sharjah, Sharjah 27272, United Arab Emirates; mani@sharjah.ac.ae (M.A.-A.); bsaddik@sharjah.ac.ae (B.S.); 4 Health and Safety Department, Dubai Municipality, Dubai 67, United Arab Emirates; dr_ammar_91_@hotmail.com; 5 Department of Family and Community Medicine and Behavioral Sciences, College of Medicine, University of Sharjah, Sharjah 27272, United Arab Emirates; 6 Department of Clinical Sciences, College of Medicine, University of Sharjah, Sharjah 27272, United Arab Emirates

**Keywords:** asthma, inhaler, technique, labels, United Arab Emirates

## Abstract

Background: Prevalence of asthma in the United Arab Emirates (UAE) is high, and training patients on correct inhaler technique is vital. Objectives: To assess the effectiveness of inhaler technique labels incorporating the individual technique steps in image format on the retention of correct inhaler technique for patients with asthma living in the UAE and following inhaler training; secondly to investigate the effect of inhaler technique education using self-check pictorial labels on patients’ overall asthma control. Methods: This single-blinded randomized controlled study was conducted in 2019 and followed consecutive recruitment of asthma patients visiting respiratory clinics at Rashid Hospital in Dubai. Patients were using a controller inhaler (Turbuhaler (TH), Accuhaler (ACC), or pressurized metered-dose inhaler (pMDI)). Following recruitment, patients were randomized into active group receiving educational intervention plus the inhaler label, and control group receiving educational intervention without the label. Patients were assessed at baseline and at one-month on their inhaler technique and asthma control. Results: Participants (*n* = 245; 93 = TH, 70 = ACC, 82 = pMDI) showed a significant difference between the groups at one-month for inhaler technique scores for TH (active 5.29 ± 1.86 vs. control = 24.4 ± 21.28), ACC (active = 3.99 ± 1.43 vs. control = 25.45 ± 22.57), and pMDI (active = 4.59 ± 0.10 vs. control = 120.55 ± 17.2), *p* < 0.001 for all. Asthma control for active group indicated significant improvements compared to control for TH and pMDI (*p* < 0.001 for both), but not ACC group (*p* = 0.087). Conclusions: Retention of correct inhaler technique and improved asthma control can be enhanced by using a specialized inhaler technique label in image format.

## 1. Introduction

Asthma is a chronic disease that affects the airways of the lungs and is characterized by inflammation and narrowing of the respiratory passages, in addition to recurrent bouts of shortness of breath associated with coughing, wheezing, and chest tightness [1]. An asthma attack is often in response to the exposure of reactive substances, allergies, or respiratory irritations, and varies in severity and frequency from person to person. According to the Centers for Disease Control and Prevention (CDC), 1 in 13 people have asthma, which accounts for 9.8 million physician’s office visits, 188,968 discharges from hospital inpatient care, and 1.8 million emergency department visits each year [2]. Asthma is prevalent worldwide, with over twenty-five million Americans suffering from the disease, and representing about 7.7% in adults and 8.4% in children [2]. For the United Arab Emirates (UAE), the reported prevalence of asthma is higher than that in the United States of America (USA), with a reported 8% in the adult population and 12% to 13% in children [3]. The reasons for the increasing prevalence of asthma in the UAE are not yet entirely clear, however, have been reportedly attributed to several factors including behavioral, environmental, and metabolic changing trends within the UAE [3,4].

A number of medications are used in the treatment of asthma, and those delivered by the inhalation route have been found to be the most effective [1,5,6]. Pressurized metered-dose inhalers (pMDIs) and dry powder inhalers (DPIs) are the most frequently used [4]. The DPIs, including the Turbuhaler (TH) and Accuhaler (ACC), have become increasingly popular as they avoid co-ordination problems commonly associated with pMDIs [5]. However, patients using DPIs still need to carry out a set of steps correctly to ensure the best drug delivery, as well as prepare the device for inhalation and generate the needed inspiratory flow rate of at least 30 L/min [6]. With proper training, most patients can achieve the correct inhaler technique of the pMDIs and DPIs [7], but this ability usually drops quickly within a few weeks or months, and patients revert to their incorrect use of the device [8,9]. The provision of inhaler technique labels that include the individual steps describing the technique of the different inhalers can be a successful educational strategy not only to teach patients correct inhaler technique but also to remind them when they go home of the correct steps in the technique leading to long term retention of correct use of the device [10,11].

Pharmacists are uniquely positioned to deliver a service to asthma patients that not only optimizes their inhaler technique demonstration skills but ensures the sustainability of their acquired skills long term [12]. Reasons for this fact is that pharmacists are always available in their community pharmacy which is easily accessible by asthma patients at all time, secondly, pharmacists are the healthcare professional that tend to see the patients before they take their inhaler and go home, and finally, pharmacists dispense the repeat prescriptions of asthma patients’ inhalers giving them the sole chance to reassess and reeducate on inhaler technique. The inhaler technique labels using only text have been already incorporated within a service delivered to asthma patient by pharmacists which proved a success in clinical and humanistic outcomes over both short term and long term periods [10,11]. However, images can replace the text in the inhaler technique labels adding other benefits besides reminding patients of the right technique steps taught during their inhaler technique education. Images can be easier to see than text words which could make it easier for patients to note.

To date, no study has assessed the effectiveness of using inhaler technique labels incorporating the correct technique steps in image format on patients’ long term inhaler technique skills. The aim of this study was to assess the effectiveness of inhaler technique labels incorporating the individual technique steps in image format on the retention of correct inhaler technique for patients with asthma living in the UAE and following inhaler training; secondly to investigate the effect of inhaler technique education using self-check pictorial labels on patients’ overall asthma control one-month post-intervention.

## 2. Results

### 2.1. Basic Demographic Information for the Active and Control Groups

**Figure 1:**
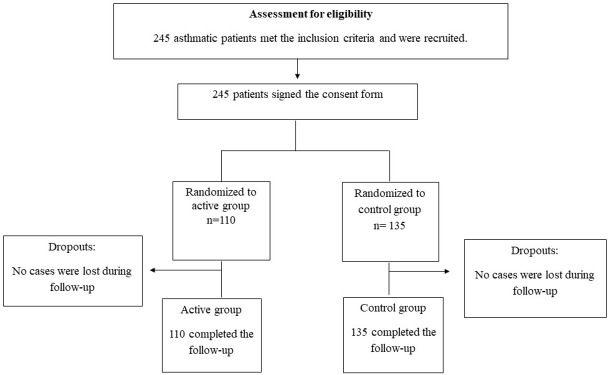
Consort diagram showing participants’ recruitment and retention during the study period.

Asthma patients (*n* = 245) were enrolled in this study and grouped based on the controller inhaler they were using at the stage of recruitment (93 TH, 70 ACC, 82 pMDI) (Figure 1). The mean age of patients was 46.7 ± 17.6 years, and 66.1% of them were females. No statistically significant differences were found between the groups with regards to their demographic and baseline characteristics (Table 1).

Education and counselling on the use of asthma inhaler devices was received by the majority of study sample. The vast majority (99.2%) received previous education on how to use their inhale, with few (8.6%) receiving verbal education, and the majority (90.6%) receiving physical demonstration of the technique (Table 2).

Many of the patients did not believe in complementary treatments for asthma management, as many (93.1%) did not use them, nor believe in their efficiency. No statistically significant differences were reported regarding complementary treatment use between the different inhaler groups (Table 3).

### 2.2. Inhaler Technique Assessment for the Active and Control Groups

Patients using the three inhalers were randomized into active and control groups, receiving the different interventions (Table 4). Results showed that significant improvements in inhaler technique demonstration skills happened over time, as fewer mistakes were demonstrated by the active group at follow-up, compared to the control groups.

A significant difference between the active and control groups was found with regards to the mean in inhaler score (lower scores indicate better inhaler technique) at follow up for the TH (active = 0.48 ± 0.67, control= 2.2 ± 1.2), ACC (active= 0.36 ± SD 0.67, control = 2.3 ± 0.78), and pMDI (active= 0.414 ± 0.68, control= 1.85 ± 1.26), which was not the case at baseline TH (active = 2.69 ± 0.81, control = 3.51 ± 1.17), ACC (active= 3.1 ± 1.02, control = 3.6 ± 1.0), and pMDI (active = 2.93 ± 1.19, control = 2.92 ± 1.33).

### 2.3. ACT Assessment

The distribution of the ACT scores for the active and control groups indicate that significant improvements in the number of patients allocated into the well-controlled asthma category group based on their score (ACT score from 20–25) for the TH and pMDI groups (*p* < 0.001 for both), but not the ACC group (*p* = 0.087) (Table 5).

Chi-square test was used in the analysis comparing the different groups. 

### 2.4. Factors Associated with Inhaler Technique Scores and ACT Scores at Follow-Up

Multiple regression modeling showed that for the dependent variable, inhaler technique score change across the study indicates that the study group type, being a male, and being a pMDI user were highly associated with higher inhaler technique improvements (Table 6 and Table 7)**.**

Table 8 displays the results of the ordinal logistic regression model for the ACT score of the one month follow up. The odds ratios in this table show the magnitude of the association and their corresponding *p*-values, indicating whether the association was statistically significant or not by using the cut-off values of 0.05. Well-controlled asthma was significantly associated with the study group type—active group (OR 5.83; 95% CI 3.09–11), and those with 5–11 years onset of asthma (OR 3.035% CI 1.65–5.56).

### 2.5. PEF and FEV Outcomes

For the active group, a significant difference was noted for the PEF outcomes between baseline (266.15) and end of study (332.25) with a mean difference of 66.10 (*p* < 0.001). The results were similar for the FEV (baseline = 71.54; follow up, *p* < 0.001). As for the control group, no significant differences were noted for the PEF nor FEV outcomes (Table 9).

## 3. Discussion

This study is the first to demonstrate that using inhaler technique labels incorporating images presenting each step in the technique is successful in maintaining correct inhaler technique one month following asthma patients’ education by pharmacists. In addition, significant improvements in clinical outcomes presented via patients’ improved ACT, FEV1, and PEF scores resulted due to this feasible education and improved inhaler technique skills.

Several previous studies showed that the patient’s inhaler technique can be corrected by a variety of educational methods, and that technique skills drop with time following education. For example, de Blaquiere and colleagues showed that, of the 62 patients who had chronic lung disease and incorrect inhaler technique, 79% were able to achieve correct technique following training, while only 55% maintained correct technique to have correct technique two months following the education [8]. Van der Palen and colleagues showed that out of the 148 patients with COPD, the proportion with correct essential technique increased from 60% at baseline to 100% after training, to fall again to 75% with the correct technique after 5 to 6 months [9]. Pothirat and colleagues reported that out of the 103 elderly patients with COPD, 41% had correct technique at baseline, improving to 100% with the correct technique after training, with only 51% showing correct technique after one month of training [10]. Basheti et al. also showed that after education at baseline, delivered by a pharmacist to 116 patients, the correct technique was demonstrated by 85% of active TH users and 96% of active ACC users; a significant difference in the proportion users dropped after six months (TH 50%, ACC 79%) [12]. Hence, feasible methods to retain correct inhaler technique skills over time amongst asthma patients are needed. This study came to answer that need in the literature of correct inhaler use. Although previous studies have proven the success of using inhaler technique labels when attached to patient’s inhalers [11], this study came to add a new variable to this approach, as technique steps shown as images instead of words on the attached labels. This came to resolve an anecdotal comment representing a complaint by patients who did not find it easy to read the words on the inhaler-attached label. This study has shown replacing the technique wording with images is successful and could be easier for patients to note.

A significant increase in the overall proportion of well-controlled asthma patients (ACT score 20–25) was detected among the study patients who received the intervention. Statistical modelling also confirmed that in this study, being in the active group, being a male, and using the pMDI as the controller device were the factors strongly associated with correct inhaler technique skills. Similar interventions also led previously to improved clinical outcomes [10,11,12], indicating that improving patients’ inhaler technique is expected to result in patients’ improved lung function [7,13,14]. In the present study, improved in inhaler technique one month following education led to improved lung function amongst the three device users, the TH, ACC and pMDI. Of noteworthy, the distribution of the ACT scores for the active and control groups indicated significant improvements in the number of patients allocated into the well-controlled asthma category group for the TH and pMDI groups but not the ACC group; such outcomes call for further investigations to unveil the reasons behind this outcome, and if the drug dose yielded by ACC is less dependent on correct technique when compared to the other two devices. The fact that a higher proportion of ACC users (61.3%) were categorized in the uncontrolled asthma category compared to TH (31.4%) and pMDI (34.0%) could also have played a role. Additionally, PFM scores significantly improved between baseline and follow up for both the control and active groups, even though the mean difference in mean scores was higher in the active group. This suggests that both verbal instruction and personalized labels incorporating technique images are effective in educating patients in using their inhalers, however, the use of the personalized labels showed higher mean differences in PFM and FEV scores between baseline and follow-up, highlighting it to be a better method to use than verbal instruction alone.

The role of the pharmacists demonstrated in the application of the inhaler technique labels via a simple educational intervention was solidified through the outcomes of this study. Once a patient leaves the pharmacy, their primary source of information about inhaler technique is the leaflet packaged with the inhaler. However, patients with asthma rarely read these, and often throw them away [11]. Hence, some studies trialed take-home materials, such as written instructions and videos to help patients maintain their inhaler technique skills long-term [12,15]. Van der Palen and colleagues, for example, provided patients with a copy of the inhaler technique checklist marked with their errors [9]. However, patients must remember and choose to use such supplementary material for it to provide any benefit. Basheti et al. used an inhaler technique label, being attached to the device itself, and could be seen every time the patient uses it, which led to significantly better inhaler technique with time [10]. In this study, such an approach was repeated, but as many patients do not find it easy to read printed checklists, images were proposed by the research team as a useful alternative. Images work better at reminding patients of the right technique steps taught during their inhaler technique education. In addition, the printed checklist labels led to improvements in asthma symptom control with no significant difference reported; only improved inhaler technique scores and lower reliever use were found [10]. The labels used in this study, incorporating images instead of text, led to significant improvements in asthma control for TH and pMDI (but not for the ACC group) in addition to improvements in inhaler technique indicating better outcomes. The pharmacist, being the last healthcare professional to see the patient before they go home to use their inhaler, is found in an ideal place to use these labels and deliver the educational session to the patient. One of the important facts noted in this study is that no participant drop-outs happened, which is not the common case in similar trials. This could indicate that the participants appreciated the role of the pharmacist delivered through this unique service and hence came back to the follow-up visit.

This study comes with few limitations. Patient’s asthma control status was not considered as an inclusion or exclusion criteria in this study. Future studies could consider adding asthma control status to the exclusion criteria to exclude patients who had severely uncontrolled asthma where improving inhaler technique may not result in the foreseen benefits. The different sample sizes recruited for the different inhalers included in this study presents a limitation as well. This was hard to avoid in this current study because of the different proportion of TH, ACC, and pMDI users visiting the respiratory clinics at which the study was conducted. Furthermore, a longitudinal assessment over a longer period of time (for example 3, 6, and 12 months) of this intervention would add strength and value to the study outcomes.

## 4. Materials and Methods

This single-blinded randomized parallel-group active-controlled study was conducted in 2019 and approved by Dubai Scientific Research Ethics Committee (DSREC), Dubai Health Authority (DSREC-10/2018-2019), and from the Research Ethics Committee, University of Sharjah (REC-17-01-29-04). Consecutive recruitment of asthma patients visiting respiratory clinics at Rashid Hospital in Dubai and who were using a controller device medication (TH, ACC, or pMDI) took place as patients were approached by the researcher. Those patients who agreed to participate and were aged 14 years and over, were using a controller medication (inhaled corticosteroid (ICS) with or without long-acting β2-agonist) via a TH, ACC, or pMDI, and who were using the same medication and dose for over one-month prior to study enrollment were included in the study.

All patients signed informed consents before study entry. Patients were informed that the study was on asthma management in general, with no mention of inhaler technique assessment or education took place. Patients were excluded from the study if they did not self-administer their inhaler medication, were not able to return for all visits, were involved in other clinical studies, or were not able to speak or understand Arabic.

Following recruitment, patients were randomized (via a predetermined randomization number list was designed through a computer-generated randomization program (www.randomization.com (accessed on 12 October 2020)) into active (patients who received the educational intervention plus the inhaler label) and control (patients who received the educational intervention but without the inhaler label) groups. Participants then received the intervention session (based on their grouping) from the researcher (a pharmacist) at the pharmacy close to the respiratory clinic they were recruited from.

### 4.1. Baseline Assessments

At baseline, demographics data, asthma medications, age at diagnosis of asthma, and reliever use in the previous month were collected.

Asthma symptom control was assessed using the published Arabic translation of the 5-item asthma control test (ACT, range 5–25) [15]. Patients were assessed on the ACT on admission (baseline) and one month later. The ACT is a validated questionnaire [13] often used to evaluate asthma control in clinical care settings, reflecting the patient’s status over the previous four weeks.

Patients’ inhaler technique with their controller device (TH, ACC, or pMDI) was assessed by a trained researcher using placebo inhalers provided by AstraZeneca Pharmaceuticals and GlaxoSmithKline and validated inhaler technique checklists translated into Arabic [14,16]. The checklist for each device consisted of nine. For the three study devices (TH, ACC and pMDI), every incorrect step perfumed by the patient was given one point, with the highest score (9) transferred to a percentage (ranging from 0% to 100%) with higher values indicating worse technique.

### 4.2. Intervention Delivered to the Active and Control Groups

Following baseline assessment, patient’s inhaler technique was optimized for both active and control groups, using the “Show and Tell” inhaler technique counselling service. In this specialized “Show and Tell” inhaler technique counseling service, the researcher went through each step on the device-specific checklist with the patient in Arabic, to describe and demonstrate correct use. This cycle of assessment and counseling was repeated up to three times if necessary, until the patient demonstrated the correct technique on all steps.

For the active group patients only, the researcher used a highlighter pen to identify all incorrect images representing the incorrect steps from the patient’s initial (pre-education) assessment on an “Inhaler Technique Images Label” (Figure S1), which was preprinted with the relevant device checklist. The researcher attached the highlighted label to the patient’s controller inhaler (not the box), without covering any essential information.

Spirometer and peak expiratory flow (PEF) testing were completed for each patient in the active and control groups. Via the spirometer, forced expiratory volume (FEV) readings measured in units of liters per minute (L/min) were recorded. As for the PEF, a peak flow meter was used (PFM; a small, hand-held device used to monitor a person’s ability to breathe out air, measuring his/her airflow through the bronchi and thus the degree of obstruction in the airways). To use the spirometer or the peak flow meter, the participants were asked to forcefully blow into the device. The spirometer provided the measurements automatically, assessing the forced air out from the patient’s lungs (in liters per minute). Similarly, with the peak flow meter, the assessment was recorded as the indicator on the device moved in response to the participant’s exhalation, providing a reading on a numbered scale.

### 4.3. Statistical Analysis

Data were analyzed using the IBM SPSS statistical package version 23. Qualitative variables were summarized using frequencies and percentages. Mean and standard deviations were used to summarize continuous variable. The Chi-square and Fisher Exact tests were used to compare differences in proportions of qualitative variables. Paired and independent-sample t-test were used to compare differences in quantitative variables. A *p*-value < 0.05 was used to test for statistical significance.

Multiple linear regression analysis was performed to investigate the association between inhaler technique scores and risk factors (study randomization group (active vs. control), patient’s gender (male vs. female), types of inhaler (TH, ACC, or pMDI)).

The stepwise method was used for variable selection and model building. Ordinal logistic regression analysis was used to investigate the association between ACT mean scores and risk factors (study randomization group (active vs. control), patient’s gender (male vs. female), type of inhaler (TH, ACC, or pMDI)), age, smoking status (smoker, nonsmoker, ex-smoker), onset of asthma (≥12 years, 0–4 years, 5–11 years, education (yes, no), complementary treatment use (yes, no), BMI, PEF, FEV.

### 4.4. Sample Size Calculations

Sample size determination was based on the primary outcome variable of inhaler technique scores improvement pre- and post-education based on our previous work in this area [10,12]. In order to detect a significantly different change in inhaler technique score of 1-point difference, with a significance level of 5%, and power of 80%, with the standard deviation of the change being 1.4 points [12], a sample size of 15 patients for each type of inhaler used (TH, ACC, and pMDI) needed to be recruited into this pre-post designed study. Accounting for a dropout rate of 20%, a sample size of 54 patients would be required. The sample size was increased to 245 to allow for analysis of factors relating to change in ACT score.

## 5. Conclusions

This study showed that retention of correct inhaler technique can be enhanced by attaching a personalized label incorporating images of the technique steps highlighting the patient’s errors to their inhaler. The use of this inexpensive label led to clinical improvements for asthma patients, including ACT scores, PEF and FEV outcomes. The labels represent an innovative, inexpensive, feasible, scalable intervention that increases the clinical efficiency of inhaler training with the potential to extend the resulting improvement in asthma clinical outcomes. The study also highlights the role of the pharmacist delivering the intervention described in this study. Recent asthma guidelines emphasize the importance of checking and correcting inhaler technique skills for asthma patients at every opportunity, knowing that poor inhaler technique is a major problem contributing to the risk of uncontrolled asthma. Pharmacists teaching patients on correct inhaler use can result in the correct use of their inhalers but maintaining the correct technique demonstration skills over time has been a challenge till today and that is where the value of the personalized label incorporating images of the technique steps to the inhaler is shown. Improving patient’s TH and pMDI technique led to a significantly higher proportion of patients in the well-controlled asthma category, which was not the case with the ACC. Further investigational studies are sought to clarify this outcome.

## Figures and Tables

**Table 1:** Demographic information for the different inhaler groups participants (*n* = 245).

Parameters	All Patients (*n* = 245)							
	All		TH		ACC		pMDI	
	Active (*n* = 110)	Control (*n* = 135)	Active (*n* = 42)	Control (*n* = 51)	Active (*n* = 39)	Control (*n* = 31)	Active (*n* = 29)	Control (*n* = 29)
Age								
mean ± SD	43.3 ± 16.5	49.6 ± 18	47 ± 15.2	49.7 ± 16.3	44.9 ± 17.6	46.6 ± 15.8	35.6 ± 15	35.6 ± 15
Height (cm)								
mean ± SD	160.4 ± 12.4	162 ± 10	158.7 ± 15.7	162 ± 10	159.2 ± 9.8	161.4 ± 10	164.6 ± 9.2	164.6 ± 9.2
Weight (Kg)								
mean ± SD	77.6 ± 18.8	79.4 ± 17.4	78.2 ± 19.3	77.6 ± 17.7	75.5 ± 18.8	82.3 ± 16.3	79.5 ± 18.4	79.5 ± 18.4
BMI								
mean ± SD	30.3 ± 7.7	30.4 ± 7	30.4 ± 7	29.6 ± 7.4	30.7 ± 8.6	31.7 ± 7	29.6 ± 7.5	29.6 ± 7.5
Gender								
Male *n* (%)	36 (43.4%)	47 (56.6%)	13 (40.6%)	19 (59.4%)	9 (52.9%)	8 (47.1%)	14 (41.2%)	14 (41.2%)
Female *n* (%)	74 (45.7%)	88 (54.3%)	29 (47.5%)	32 (52.5%)	30 (56.6%)	23 (43.4%)	15 (31.3%)	15 (31.3%)
Smoking status								
Never *n* (%)	92 (46.2%)	107 (53.8%)	36 (48.6%)	38 (51.4%)	34 (56.7%)	26 (43.3%)	22 (33.8%)	22 (33.8%)
Current *n* (%)	12 (48%)	13 (52%)	4 (57.1%)	3 (42.9%)	4 (57.1%)	3 (42.9%)	4 (36.4%)	4 (36.4%)
Ex-smoker *n* (%)	6 (28.6%)	15 (71.4%)	2 (16.7%)	10 (83.3%)	1 (33.3%)	2 (66.7%)	3 (50%)	3 (50%)
Onset of asthma								
0–4 years *n* (%)	2 (50%)	2 (50%)	0 (0%)	0 (0%)	1 (100%)	0 (0%)	1 (33.3%)	1 (33.3%)
5–11 years *n* (%)	54 (55.7%)	43 (44.3%)	19 (55.9%)	15 (44.1%)	17 (53.1%)	15 (46.9%)	18 (58.1%)	18 (58.1%)
≥12 years *n* (%)	54 (37.5%)	90 (62.5%)	23 (39%)	36 (61%)	21 (56.8%)	16 (43.2%)	10 (20.8%)	10 (20.8%)

TH, Turbuhaler inhaler; ACC, Accuhaler inhaler; pMDI, pressurized metered-dose inhaler; BMI, body mass index; SD, standard deviation, *n* (%), frequency (percentage).

**Table 2:** Advice and education received on asthma inhaler device use as reported by patients in the three study groups.

Parameters	All Patients (*n* = 245)							
	All		TH		ACC		pMDI	
	Active (*n* = 110)	Control (*n* = 135)	Active (*n* = 42)	Control (*n* = 51)	Active (*n* = 39)	Control (*n* = 31)	Active (*n* = 29)	Control (*n* = 53)
Receiving education on how to use the inhalers Yes	110(45.3%)	133(54.7%)	42(45.2%)	51(54.8%)	39(55.7%)	31(44.3%)	29(36.3%)	51(63.8%)
Education method received by patient on inhaler technique (*n* = 243)								
Verbal information	6 (28.6%)	15 (71.4%)	3 (37.5%)	5 (62.5%)	1 (50%)	1 (50%)	2 (18.2%)	9 (81.1%)
Written information	0 (0%)	0 (0%)	0 (0%)	0 (0%)	0 (0%)	0 (0%)	0 (0%)	0 (0%)
Physical demonstration	104 (46.8%)	118 (53.2%)	39 (45.9%)	46 (54.1%)	38 (55.9%)	30 (44.1%)	27 (39.1%)	42 (60.9%)
Being provided with a plan on asthma management (*n* = 245) Yes	0 (0%)	9 (100%)	0 (0%)	2 (100%)	0 (0%)	1 (100%)	0 (0%)	6 (100%)

TH, Turbuhaler inhaler; ACC, Accuhaler inhaler; pMDI, pressurized metered-dose inhaler BMI, body mass index; SD, standard deviation, *n* (%), frequency (percentage).

**Table 3:** Complementary treatment information for the different study inhaler groups (*n* = 245).

Parameters	All Patients (*n* = 245)							
	All		pMDI		TH		ACC	
	Active (*n* = 110)	Control (*n* = 135)	Active (*n* = 29)	Control (*n* = 53)	Active (*n* = 42)	Control (*n* = 51)	Active (*n* = 39)	Control (*n* = 31)
**Complementary treatment use**								
Yes	4 (23.5%)	13 (76.5%)	0 (0%)	8 (100%)	0 (0%)	8 (100%)	0 (0%)	1 (100%)
No	106 (46.5%)	122 (53.5%)	29 (39.2%)	45 (60.8%)	29 (39.2%)	45 (60.8%)	39 (56.5%)	30 (43.5%)
**Believe that complementary treatment works in managing patients’ asthma**								
Yes	4 (23.5%)	13 (76.5%)	0 (0%)	8 (100%)	4 (50%)	4 (50%)	0 (0%)	1 (100%)

TH, Turbuhaler inhaler; ACC, Accuhaler inhaler; pMDI, pressurized metered-dose inhaler; BMI, body mass index; SD, standard deviation, *n* (%), frequency (percentage).

**Table 4:** Inhaler technique scores for patients in the active and control groups.

	Inhaler Technique Scores						
	Active (*n* = 110)			Control (*n* = 135)			
Time of Assessment	Mean in % 95% CI			Mean in % 95% CI			*p* Value
All							
Baseline assessment	32.02	29.65	34.4	36.79	34.65	38.93	0.004
One month follow up	4.646	2.621	6.672	23.13	21.29	24.96	<0.001
TH							
Baseline assessment	29.89	26.40	33.39	38.99	35.83	42.17	<0.001
One month follow up	5.29	1.86	8.73	24.4	21.28	27.52	<0.001
ACC							
Baseline assessment	33.90	30.27	37.53	40.50	36.43	44.57	0.018
One month follow up	3.99	1.43	6.55	25.45	22.57	28.32	<0.001
pMDI							
Baseline assessment	32.57	27.3	37.83	32.49	28.6	36.4	0.983
One month follow up	4.59	0.10	9.1	20.55	17.2	23.87	<0.001

Abbreviations: pMDI, pressurized metered-dose inhaler; TH, Turbuhaler inhaler; ACC, Accuhaler inhaler; BMI, body mass index; SD, standard deviation, SD; standard deviation; *p* < 0.05 Significance; 95% CI confidence interval. Independent sample t test was used to compare between active and control groups.

**Table 5:** Assessment of asthma control test (ACT) for both active and control groups.

	Asthma Control Score						
	Active (*n* = 110)			Control (*n* = 135)			
Time of Assessment	20–25	16–19	<16	20–25	16–19	<16	*p* Value
	*n* (%)	*n* (%)	*n* (%)	*n* (%)	*n* (%)	*n* (%)	
	Well Controlled Asthma	Intermediately Controlled Asthma	Uncontrolled Asthma	Well Controlled Asthma	Intermediately Controlled Asthma	Uncontrolled Asthma	
All							
Baseline assessment	11 (10%)	42 (38.2%)	57 (51.8%)	35 (25.9%)	47 (34.8%)	53 (39.3%)	0.005
One month follow up	95 (86.4%)	9 (8.2%)	6 (5.5%)	69 (51.1%)	40 (29.6%)	26 (19.3%)	<0.001
pMDI							
Baseline assessment	2 (6.9%)	11 (37.9%)	16 (55.2%)	15 (28.3%)	20 (37.7%)	18 (34.0%)	0.046
One month follow up	26 (89.7%)	1 (3.4%)	2 (6.9%)	24 (45.3%)	17 (32.1%)	12 (22.6%)	<0.001
TH							
Baseline assessment	6 (14.3%)	10 (23.8%	26 (61.9%)	17 (33.3%)	18 (35.3%)	16 (31.4%)	0.010
One month follow up	37 (88.1%)	3 (7.1%)	2 (4.8%)	27 (52.9%)	14 (27.5%)	10 (19.6%)	0.001
ACC							
Baseline assessment	3 (7.7%)	21 (53.8%)	15 (38.5%)	3 (9.7%)	9 (29%)	19 (61.3%)	0.110
One month follow up	32 (82.1%)	5 (12.8%)	2 (5.1%)	18 (58.1%)	9 (29%)	4 (12.9%)	0.087

Abbreviations: pMDI, pressurized metered-dose inhaler; TH, Turbuhaler inhaler; ACC, Accuhaler inhaler; BMI, body mass index; SD, standard deviation; *p* < 0.05 Significance; 95% CI confidence interval.

**Table 6:** Selecting the set of factors that jointly influence the use of inhaler technique for study participants (*n* = 245).

Independent Variables	Inhaler Technique Score			
	B	95% CI		*p* Value
Education group (Ref. Control)				
Active	−18.48	−21.21	−15.75	<0.001
Gender (Ref. Female)				
Male	2.47	−1.28	6.24	0.196
Age	0.149	0.049	0.249	0.004
Smoking status (Ref. Smoker)				
Never	−6.13	−10.64	−1.62	0.008
Onset of asthma (≥12 years)				
0–11 years	−6.14	−9.68	−2.59	0.001
Education (Ref. no)				
Yes	−13.05	−32.84	6.73	0.195
Complementary treatment (Ref. no)				
Yes	−1.19	−8.22	5.84	0.740
BMI	0.144	−0.100	0.389	0.245
Inhaler type (Ref.)				
pMDI	1.41	−3.136	5.963	0.541
TH	2.28	−2.145	6.702	0.311
PFM	0.005	−0.018	0.029	0.672
FEV	−0.141	−0.298	0.016	0.077
IGE	−0.001	−0.004	0.001	0.246

Abbreviations: pMDI, pressurized metered-dose inhaler; Turbuhaler inhaler; ACC, Accuhaler inhaler; BMI, body mass index; SD, standard deviation B” is the un-standardized regression coefficient.; CI, confidence interval; *p* < 0.05 Significance.

**Table 7:** Multiple liner regression model for inhaler technique-related factors.

Independent Variables	Inhaler Technique Score						
	B	S.E.	Beta	t	95% CI		*p* Value
Study randomization group (Active)	−23.05	1.56	−0.878	−14.79	−25.93	−19.52	<0.001
Gender (Male)	4.59	1.6	0.164	2.86	1.38	7.81	0.006
Type of inhaler (pMDI)	−4.03	1.82	−0.130	−2.21	−7.67	−0.38	0.031

pMDI, pressurized metered-dose inhaler. This table shows the output from a multivariable regression analysis in which inhaler technique score at 1 months was the dependent variable. “Beta” is the standardized regression coefficient. The overall fit of the model was R2 = 0.889, *p* < 0.001.

**Table 8:** Logistic regression modelling for asthma control test-related factors.

Independent Variables	Asthma Control Test Score			
	OR	95% CI		*p* Value
study randomization group (active vs. control), (Ref. Control)				
Active	5.835	3.091	11.015	<0.001
Gender (Ref. Female)				
Male	1.101	0.631	1.922	0.735
Age	0.990	0.975	1.004	0.171
Smoking status (Ref. Smoker)				
Never	1.477	0.771	2.828	0.239
Onset of asthma (≥ 12 years)				
0–4 years	0.057	0.006	0.543	0.013
5–11 years	3.035	1.658	5.557	<0.001
Education (Ref. no)				
Yes	1.425	0.119	17.089	0.780
Complementary treatment (Ref. no)				
Yes	0.573	0.236	1.394	0.220
BMI	0.994	0.960	1.029	0.738
Inhaler type (Ref.)				
TH	0.846	0.434	1.652	0.625
ACC	1.0	1.0	1.001	0.453
pMDI	0.598	0.307	1.168	0.132
PFM	1.003	0.999	1.007	0.147
FEV	1.017	0.987	1.048	0.282

Abbreviations: pMDI: pressurized metered-dose inhaler; TH: Turbuhaler inhaler; ACC: Accuhaler inhaler; BMI: body mass index; SD: standard deviation; OR: odd ratio; *p* < 0.05 Significance; 95% CI confidence interval. Numbers in bold are statistically significant.

**Table 9:** Mean scores of inhaler technique for the peak flow meter (PFM) readings and forced expiratory volume (FEV) readings.

	Time of Assessment		Paired Difference			
All (*n* = 245)	Baseline Assessment	1 Month Follow Up	Mean Difference	95% Confidence Interval of the Difference		Sig
PFM	279.85	333.12	53.26	46.83	59.68	<0.001
FEV	71.98	75.61	3.63	1.58	5.68	0.001
Control group						
PFM	296.09	334.13	38.04	32.22	43.86	<0.001
FEV	72.86	72.44	0.42	−2.63	3.47	0.783
Active group						
PFM	266.15	332.25	66.10	55.83	76.37	<0.001
FEV	71.54	78.22	6.68	4.17	9.19	<0.001

*p* < 0.05 was considered statistically significant. Paired sample *t*-test.

## Data Availability

The data presented in this study are available on request from the corresponding author. The data are not publicly available due to privacy.

## Supplementary Materials

The following are available online at https://www.mdpi.com/1424-8247/14/2/150/s1, Figure S1: An example of Inhaler (Turbuhaler (TH)) technique label incorporating images attached to the patient’s inhaler during the inhaler technique educational service.
