# Supplementary material for: Assessment of Novel Inhaler Technique Reminder Labels in Image Format on the Correct Demonstration of Inhaler Technique Skills in Asthma: A Single-Blinded Randomized Controlled Trial
Source: Pharmaceuticals (Basel). 2021 Feb 12;14(2):150. doi: 10.3390/ph14020150 (PMC7918490; doi:10.3390/ph14020150)
Supplement: Supplementary file 1 [file pharmaceuticals-14-00150-s001.pdf]

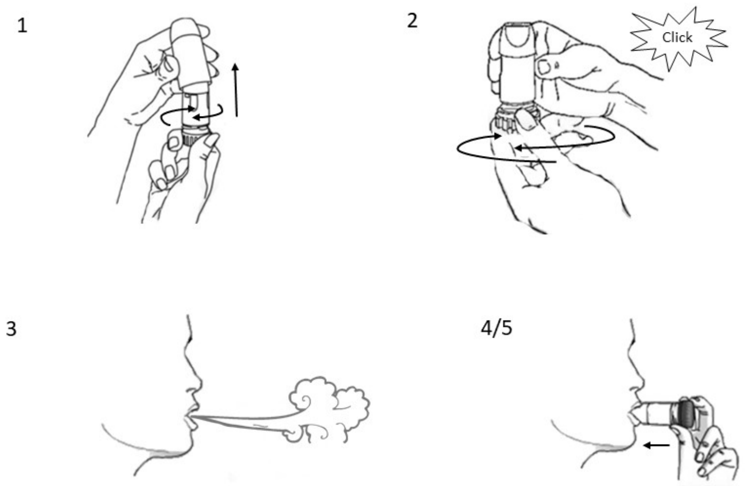

Figure S1: An example of inhaler (Turbuhaler, TH) technique label incorporating images attached to the patient's inhaler during the inhaler technique educational services.
